# A Novel Chitosan-γPGA Polyelectrolyte Complex Hydrogel Promotes Early New Bone Formation in the Alveolar Socket Following Tooth Extraction

**DOI:** 10.1371/journal.pone.0092362

**Published:** 2014-03-21

**Authors:** Hao-Hueng Chang, Yin-Lin Wang, Yu-Chih Chiang, Yen-Liang Chen, Yu-Horng Chuang, Shang-Jye Tsai, Kuo-Huang Heish, Feng-Huei Lin, Chun-Pin Lin

**Affiliations:** 1 School of Dentistry, National Taiwan University, Taipei, Taiwan; 2 Department of Dentistry, National Taiwan University Hospital, Taipei, Taiwan; 3 Department of Dentistry, Cardinal Tien Hospital Yonghe Branch, New Taipei, Taiwan; 4 Department of Chemical Engineering, National Taiwan University, Taipei, Taiwan; 5 Institute of Biomedical Engineering, National Taiwan University, Taipei, Taiwan; University Hospital of the Albert-Ludwigs-University Freiburg, Germany

## Abstract

A novel chitosan-γPGA polyelectrolyte complex hydrogel (C-PGA) has been developed and proven to be an effective dressing for wound healing. The purpose of this study was to evaluate if C-PGA could promote new bone formation in the alveolar socket following tooth extraction. An animal model was proposed using radiography and histomorphology simultaneously to analyze the symmetrical sections of Wistar rats. The upper incisors of Wistar rats were extracted and the extraction sockets were randomly treated with gelatin sponge, neat chitosan, C-PGA, or received no treatment. The extraction sockets of selected rats from each group were evaluated at 1, 2, 4, or 6 wk post-extraction. The results of radiography and histopathology indicated that the extraction sockets treated with C-PGA exhibited lamellar bone formation (6.5%) as early as 2 wk after the extraction was performed. Moreover, the degree of new bone formation was significantly higher (*P* < 0.05) in the extraction sockets treated with C-PGA at 6 wk post-extraction than that in the other study groups. In this study, we demonstrated that the proposed animal model involving symmetrical sections and simultaneous radiography and histomorphology evaluation is feasible. We also conclude that the novel C-PGA has great potential for new bone formation in the alveolar socket following tooth extraction.

## Introduction

Healing the alveolar socket following tooth extraction relieves discomfort and preserves the height of the alveolar ridge [1], [2]. Although numerous types of graft materials, such as calcium phosphate [3], hydroxyapatite [4], [5], borosilicate [6], chitosan [7], and gelatin [8], [9], have been proposed as candidates for graft materials based on the ability to promote bone healing [10], an ideal bone graft material has not been identified [1].

Hydrogels are composed of 3-dimensional hydrophilic material. The hydrophilic surface of hydrogels produces low interfacial free energy when it is in contact with body fluid and, therefore, exhibits excellent biocompatibility [11], [12]. Because of these properties, hydrogels have recently been used as drug carriers and artificial tissue scaffolds [11], [13]. Although they contain hydrophilic polymeric backbones, hydrogels are not dissolved in water when radical, chemical, or physical crosslinks are present [11], [12]. Furthermore, radical crosslinks provide a high crosslinking quality, but residual radicals may still exist in the hydrogels. These safety concerns consequently limit the use of radical crosslinks. A chemical crosslink is also unfavorable for biological applications because it requires a toxic crosslinker to achieve covalent bond formation between various polymer chains. Ionic interaction is a physical interaction, the safety and role of which in rendering polymer hydrophilic material and causing the material to exhibit a high water uptake was reported. Consequently, we chose 2 oppositely charged agents to form polyelectrolyte complex (PEC) hydrogels [11], [12].

Chitosan is obtained from the deacetylation of chitin, a naturally occurring, biocompatible polysaccharide that is abundant in the exoskeleton of numerous classes of invertebrates, including crustaceans. Chitin is a copolymer composed of N-acetyl-glucosamine and N-glucosamine subunits, the distribution of which may vary considerably between species [14]. Chitosan is a cationic polysaccharide that is insoluble in neutral or basic solutions because it possesses a slightly crystalline character [11], [12]. However, an acidic environment enables the free amino groups of chitosan to become protonated. Therefore, the molecule is soluble in low pH solutions with a positive charge. The high positive charge of chitosan permits the formation of a polyelectrolyte complex hydrogel with polyanionic species in an acidic environment [11], [12].

The antibacterial property of chitosan was reported, and chitosan was also used as a wound dressing in veterinary medicine because of its ability to accelerate the healing process [11], [12]. In addition, in vivo and in vitro studies have indicated that chitosan oligomers and chitin oligomers, originating from enzymatic degradation in a wound environment, produce stimulatory effects on macrophages. These studies have also demonstrated that the migratory activity of mouse peritoneal macrophages is significantly enhanced [12].

Produced as a capsular substance or a component of the slime envelope by members of Bacillus, the biopolymer poly(γ-glutamic acid), or γ-PGA, is a naturally occurring anionic compound composed of a homopolyamide that comprises D- and L-glutamic acid subunits crosslinked by amide bonds between the α-amino and carboxylic acid functional groups of each subunit [14]. Thus, γ-PGA is a polypeptide comprised solely of glutamate residues. Unlike chitosan, γ-PGA is water soluble [12], [13], biodegradable, nontoxic, and edible. γ-PGA also exhibits excellent tissue affinity and, consequently, has been used in biological glue and drug delivery systems. The high anionic property allows γ-PGA to form a polyelectrolyte complex hydrogel with chitosan in a biologically appropriate pH value [11], [12]. When a polycation and a polyanion are combined in aqueous solution, a polyelectrolyte complex is formed. Therefore, an equimolar complex is formed by mixing oppositely charged polyelectrolytes [15].

We developed a novel and structurally stable chitosan-γPGA PEC hydrogel (C-PGA) that contained antiseptic, biocompatible, and biodegradable properties, and maintained the hydrogel form at a biologically appropriate pH [11]. We also demonstrated that C-PGA provided adequate moisture and, thus, reduced the risk of dehydration. The animal study also indicated that wounds treated with C-PGA healed significantly faster than wounds treated with neat chitosan or given no treatment [12]. The aims of this study were to establish an animal model using radiography and histomorphology simultaneously to analyze the symmetrical sections of Wistar rats, and to evaluate the effectiveness of the novel C-PGA to promote the healing and new bone formation of the alveolar socket following tooth extraction.

## Materials and Methods

### Ethics Statement

The animal experiments performed in this study were approved by the Institutional Animal Care and Use Committee at the Medical College of National Taiwan University.

### Reagents

The γPGA (average molecular weight, 1250 kDa) was purchased from Vedan (Taichung, Taiwan). The chitosan (average molecular weight, 300 kDa) with 97% to 98% de-acetylation was purchased from G-HT (Hsinchu, Taiwan). Acetic acid (AcOH), sodium hydroxide (NaOH), and other reagents were purchased from Sigma-Aldrich (St Louis, MO, USA). The sterile, porcine-gelatin Spongostan sponge was purchased from Johnson & Johnson (New Brunswick, NJ, USA).

### Preparation of C-PGA

**Figure 1:**
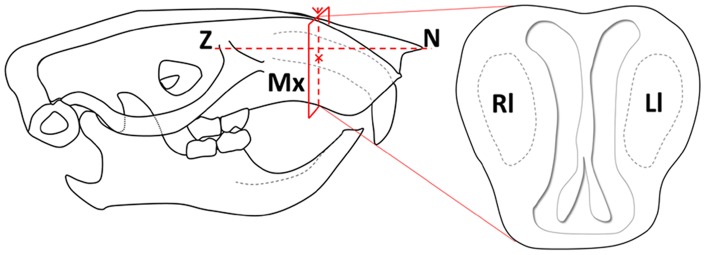
Microphotographs obtained using scanning electron microscopy (SEM) that reveal the cross sections of the chitosan/rPGA hydrogel graft with pore sizes from 60 to 250 μm. A: 80X, B: 200X.

The C-PGA grafts were prepared using a previously described method [11], [13] with a 1∶1 molar ratio of amino groups (chitosan) to carboxylic acid groups (γPGA). Chitosan powder was combined with an aqueous solution of γPGA at a chitosan-to-γPGA (weight-to-weight) percentage of 4%. The solution was mixed thoroughly and 1% acetic acid was added. Under these conditions, the chitosan powder dissolved immediately because of its protonated amino groups. The homogeneous hydrogel was subsequently formed through the complex formation between the amino group of chitosan and the carboxylic acid group of γPGA. The polymerized C-PGA was then immersed in 1 N NaOH and neutralized to pH 7 by washing it with deionized water. The C-PGA was freeze dried to form the porous matrix with a 1∶1 ratio of chitosan to γPGA (Fig. 1).

### Preparation of Neat Chitosan

The neat chitosan was prepared by using the immersion-precipitation method [11], [13]. The neat chitosan was precipitated by combining a 4% chitosan solution in 1% acetic acid with 1 N NaOH. The solidified chitosan was neutralized by washing it with deionized water and freeze drying it to form the porous matrix.

### Graft Implantation

The upper incisors of 40 male Wistar rats under general anesthesia were extracted using 0.1 mL Zoletil (Tiletamine and Zolazepam, 25 mg/mL each). Immediately after removing the teeth, one socket was treated with one test material, and the other socket was treated with another material for comparison. Thus, the extraction sockets were divided into the control alveolar socket group (no graft treatment) and the gelatin-sponge, neat-chitosan, and C-PGA treatment groups. Rats were sacrificed at 1, 2, 4, or 6 wk post-extraction for the analysis of the extraction sites. (n = 5 in each group at different sacrificed time point)

### Radiographic Examination and Grading

The rats were euthanized by asphyxiation using carbon dioxide at 1, 2, 4, or 6 wk post-extraction for radiographic and histological evaluations. Clinical radiographic examination was used to evaluate the degree of bone mineralization (DBM) in the extraction sockets during healing. Because all of the graft materials used in this study exhibited radiolucency, the relative size of the radiopaque area following surgery correlated with the degree of calcification at the extraction site. The semiqualitative grading system used for radiographically examining DBM was based on the following parameters at 10× magnification: (a) sections with no evidence of radiopacity were assigned a score of zero; (b) a radiopaque area comprising less than 25% of the overall area of the socket was assigned a score of one; (c) a radiopaque area comprising 25% to 49% of the overall area of the socket was assigned a score of 2; (d) a radiopaque area comprising 50% to 74% of the overall area of the socket was assigned a score of 3; and (e) a radiopaque area comprising 75% or more of the overall area of the socket was assigned a score of 4. Each section was scored by 2 independent examiners, and a third examiner resolved the scoring differences.

### Histological Examination and Assessment

#### Histological Assessment

**Figure 2:**
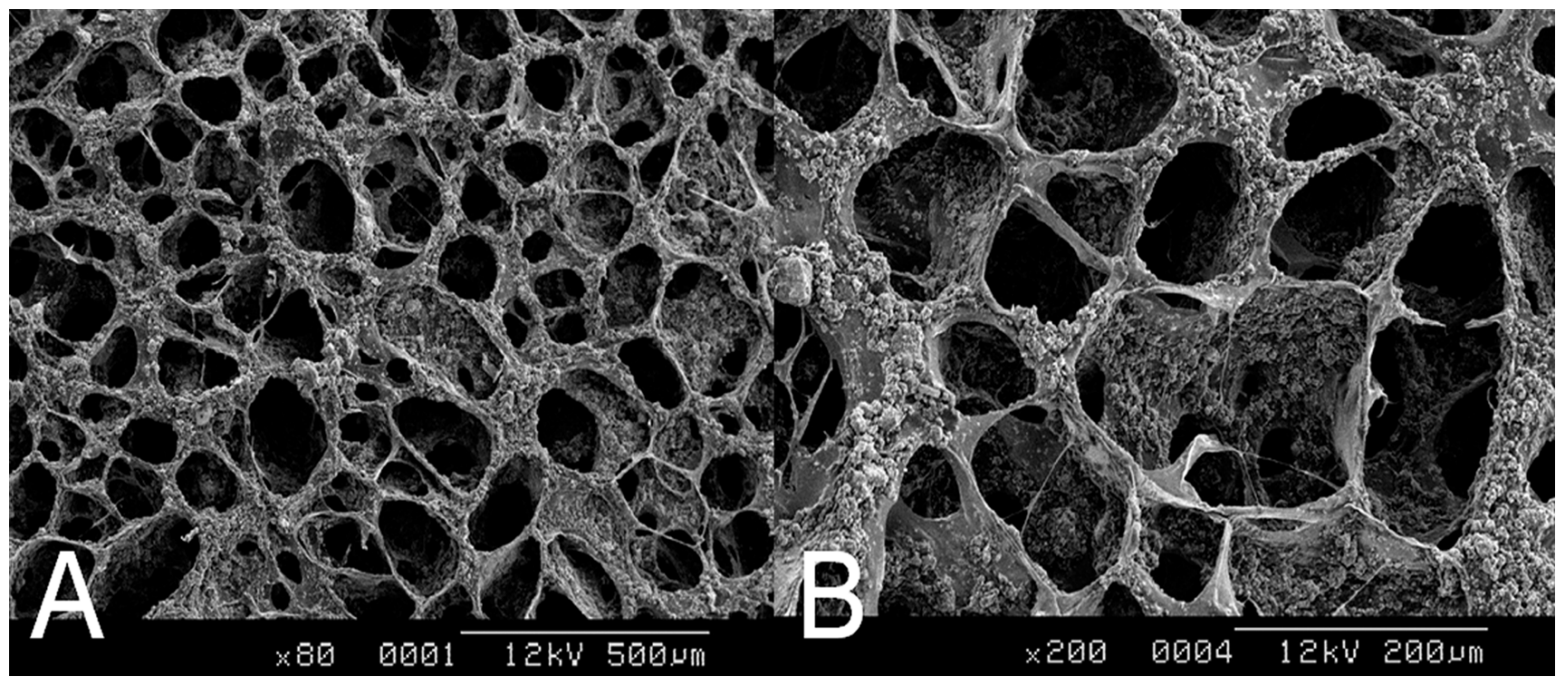
Illustration of the section plane in the rat maxilla. A reference line from the top of the zygoma (Z) to the nasion (N) was first drawn, and then the section plane lying perpendicular to the reference line and crossing through the central point was determined. The right and left extraction sockets of the upper incisors (RI & LI) are located in the maxilla (Mx) on the lateral side of the nasal cavity.

Coronary sections were made perpendicular to the anterior-posterior axis of the heads of the euthanized rats, as shown in Fig. 2. The head, including the upper jaw, was removed and was subsequently fixed in a 4% paraformaldehyde solution. After fixation, the tissues were fixed further in 10% neutral buffered formalin, decalcified in 5% formic acid, embedded in paraffin, sectioned at 5-μm increments, and stained with hematoxylin and eosin. All of the sections were evaluated using a Zeiss Primo Star microscope (Zeiss, Heidelberg, Germany) for the presence or absence of grafts and bone regeneration. One section from each socket was selected for further analysis based on the largest area of DBM or the greatest amount of bone regeneration observed. Graft tissues were examined histologically for evidence of osteoinduction using a previously described qualitative scoring method [16]. In addition, histomorphometry was used to assess the amount of new bone formation, the size of individual ossicles, the area of each ossicle occupied by marrow, and the amount of residual graft material. The results of the qualitative scoring were categorized as follows: (a) sections exhibiting no evidence of DBM or new bone growth were scored as 0; (b) sections in which DBM was observed, but no new bone or cartilage was present, were assigned a score of 1; (c) sections in which a single ossicle (marrow space surrounded by new bone and DBM) was observed were assigned a score of 2; (d) sections in which 2 or more ossicles were present were assigned a score of 3; and (e) sections containing highly osteoinductive grafts, for which 70% of the slide at 10× magnification was covered with an ossicle, were assigned a score of 4. For the qualitative scoring system, each section was evaluated by 2 independent examiners. A third examiner resolved any differences in scoring. Sections with a histological score of 0 were not included in the calculations of the average score.

#### Histomorphological analysis

Decalcified and non-decalcified (ground) sections were prepared using previously described methods [17], [18]. The histological examination was performed using a Zeiss Primo Star microscope (Zeiss, Heidelberg, Germany) equipped with an iCAM-500 CMU imaging system (CMU, Taipei, Taiwan).

The proportions of woven bone, lamellar bone, non-mineralized structures (residual tissue), and bone remnants (debris) were enumerated in the ground sections. A morphometric point-counting procedure was performed using a lattice consisting of 100 light points superimposed over the tissue of decalcified sections at 1000× magnification, as described previously [19]. The tissue within the healing socket was analyzed for the presence of osteoblasts, fibroblast-like mesenchymal cells, adipocytes, erythrocytes, granulocytes, lymphocytes, plasma cells, monocytes and macrophages (M), vascular structures, mineralized tissue components, and unidentified structures (residual tissue).

### Statistical analysis

The results of the analysis of both the continuous and categorical variables are presented as the mean and standard deviation (mean ± SD). Differences in the measured properties between the study groups were evaluated using a one-way analysis of variance with 95% confidence intervals. Differences with a P value less than 0.05 were considered statistically significant.

## Results

### Radiographic and histopathological findings for the control group

**Figure 3:**
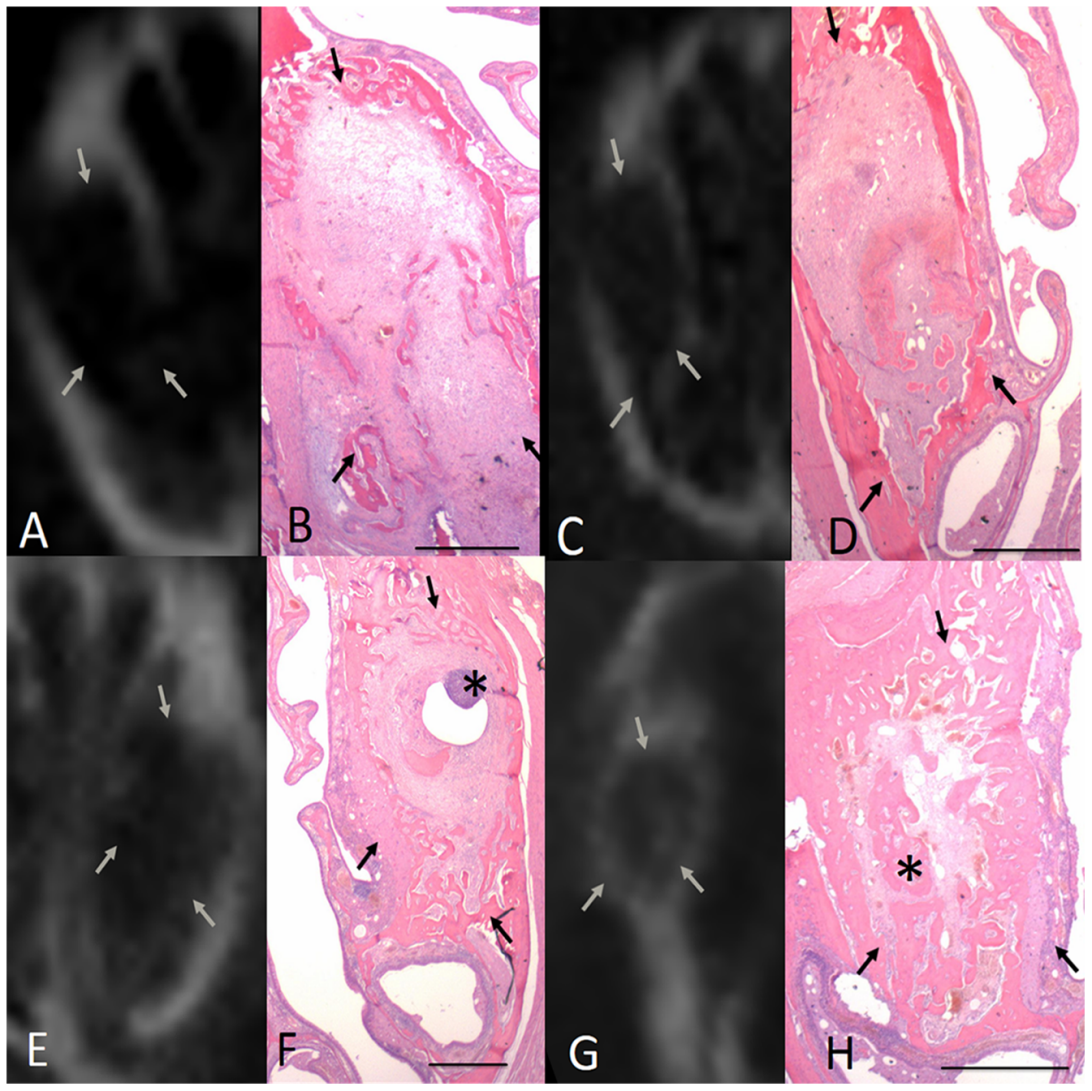
Radiographic and histopathological findings regarding socket healing in the control group at various time points post-extraction. A. At 1 wk post-extraction, no radiographically detectable calcification was visible in the extraction socket. B. Histologically, the extraction socket was filled with a provisional matrix consisting of collagen fibrils and vascular structures. C. After 2 wk, no radiographically detectable calcification was found. D. The extraction socket was filled with provisional spongiosa that was rich in vascular structures and fibroblast-like cells. E. After 4 wk, some radiographically detectable calcification was present. F. The extraction socket contained trabecular bone along the socket wall. Scattered inflammatory cell infiltration was observed in the sockets (*). G. After 6 wk, some radiographically detectable calcification was present in the control extraction sockets. H. The extraction socket contained trabecular bone from the peripheral socket wall to the central portion of the socket. Large areas of the newly formed bone were characterized by the formation of primary and secondary osteons (*). Arrows indicate the length of the extraction socket (scale bar for B, D, F, and H is 1 mm).

The radiographic and histopathological findings regarding socket healing in the control group are shown in Fig. 3. At 1 and 2 wk post-extraction, the control group sockets were filled with granulation or connective tissues, and displayed prominent inflammatory cell infiltration consisting mainly of granulocytes, whereas no new bone was visible in the socket. At 4 wk post-extraction, a small amount of trabecular bone formation was visible along the walls of the control group sockets. However, the central region of the sockets remained filled with connective tissues, and no significant inflammatory cell infiltration was observed. At 6 wk post-extraction, a significant amount of newly formed bone was present between the periphery and the central region of the control alveolar sockets.

### Radiographic and histopathological findings for the gelatin sponge group

**Figure 4:**
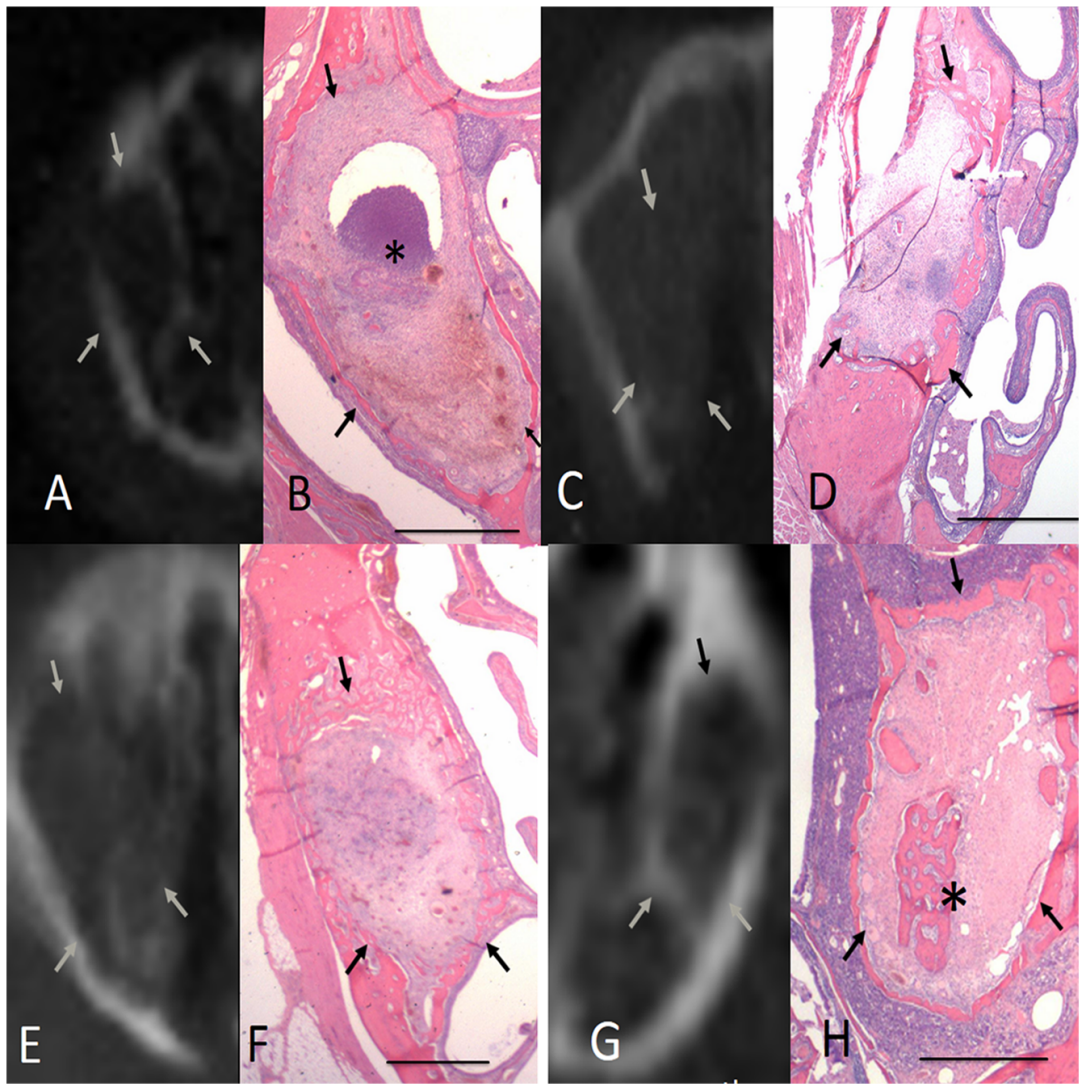
Radiographic and histopathological findings regarding socket healing in the gelatin sponge group at various time points post-extraction. A. After 1 wk, no radiographically detectable calcification occurred in the extraction socket. B. Histologically, the socket contained prominent inflammatory cell infiltration, consisting mainly of granulocytes (*), without newly formed bone. C. After 2 wk, no radiographically detectable calcification was present. D. The socket contained abundant granulation tissues with prominent inflammatory cell infiltration. E. After 4 wk, some radiographically detectable calcification was present along the wall of the extraction sockets. F. A small amount of trabecular bone formed in the peripheral regions of the extraction sockets. G. After 6 wk, an increased amount of radiographically detectable calcification was present in the extraction socket. H. Some amount of trabecular bone formed in both the central (*) and peripheral regions of the extraction sockets. Arrows indicate the length of the extraction socket (scale bar for B, D, F, and H is 1 mm).

The radiographic and histopathological findings regarding socket healing in the gelatin sponge group are shown in Fig. 4. The extraction sockets that were treated with the gelatin sponge at 1, 2, and 4 wk exhibited characteristics that were similar to those of the control group. At 6 wk, a small amount of trabecular bone formation occurred in the central region, and additional trabecular bone formation occurred in the peripheral regions of the control extraction sockets. However, fibrotic tissue surrounded by inflammatory cell infiltration was also visible.

### Radiographic and histopathological findings for the neat chitosan group

**Figure 5:**
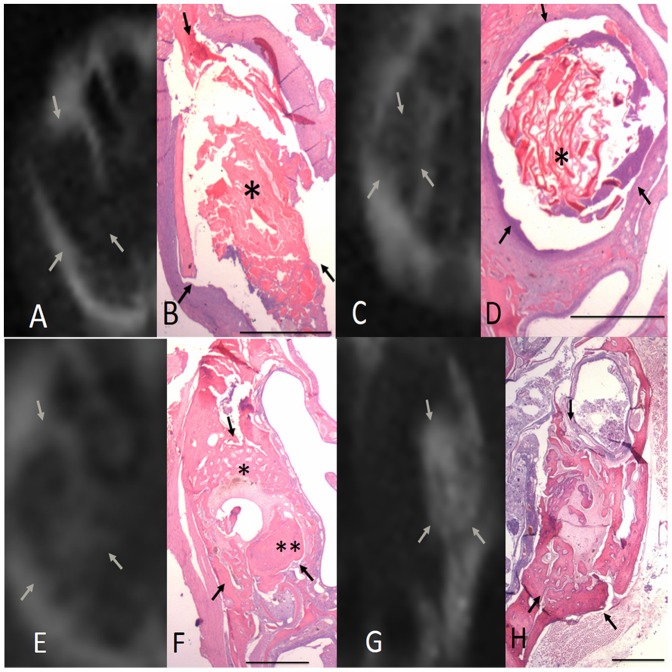
Radiographic and histopathological findings regarding socket healing in the neat chitosan group at various time points post-extraction. A. After 1 wk, no radiographically detectable calcification occurred in the extraction sockets. B. Histologically, the socket contained fragmented and degraded chitosan (*). Inflammatory cell infiltration, consisting mainly of granulocytes, was visible around the chitosan graft. Traces of woven bone formation were also observed. C. After 2 wk, no radiographically detectable calcification was present. D. The socket contained a significantly reduced amount of chitosan material, compared with the contents at 1 wk post-extraction. Residual chitosan (*) with surrounding inflammatory cell infiltration was observed in most areas of the extraction socket. E. After 4 wk, some radiographically detectable calcification was present along the wall of the extraction sockets. F. No residual graft was identified. A substantial amount of woven bone was visible in the peripheral regions of the socket (*), but spongiosa that was rich in vascular structures and some lamellar bone were observed (**). G. After 6 wk, a substantial amount of radiographically detectable calcification was present in the extraction sockets. H. Most regions of the extraction socket were occupied by newly formed, loose trabecular bone. Arrows indicate the length of the extraction socket (scale bar for B, D, F, and H is 1 mm).

The radiographic and histopathological findings regarding socket healing in the neat chitosan group are shown in Fig. 5. At 1 wk post-extraction, the chitosan was fragmented and partially resorbed in the extraction sockets. The status of inflammatory cell infiltration and new bone formation was similar to that of the control and gelatin sponge groups. At 2 wk following extraction, some residual chitosan materials remained in the extraction sockets. In addition, inflammatory cells were present and no new bone formation was visible. At 4 wk post-extraction, no residual chitosan was identified in the sockets, and trabecular bone formation was present in both of the peripheral regions and the central region of the chitosan-treated sockets. Osteons and Haversian canals were visible at a higher magnification. At 6 wk post-extraction, most of the socket regions were occupied by newly formed and loose trabecular bone.

### Radiographic and histopathological findings for the C-PGA group

**Figure 6:**
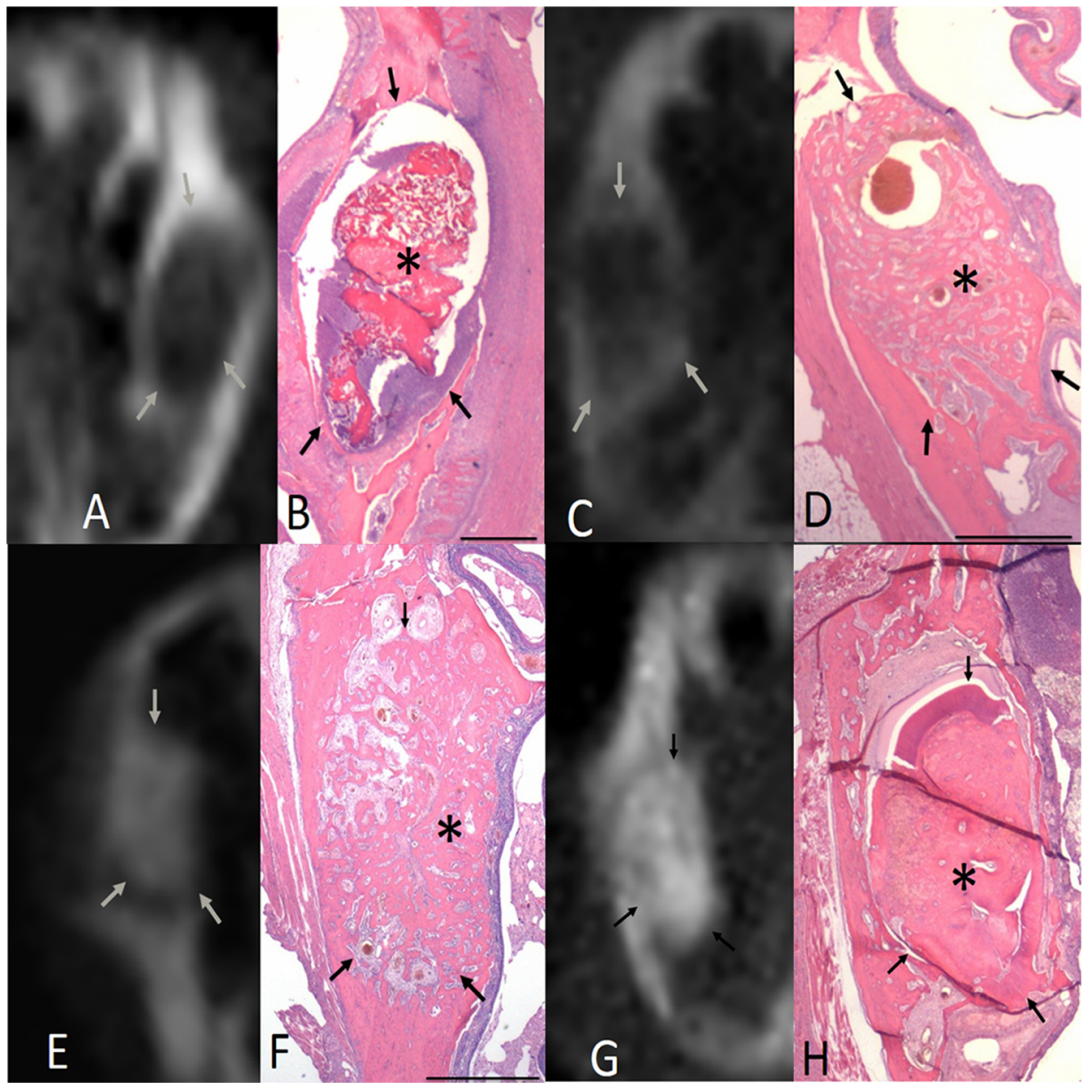
Radiographic and histopathological findings regarding socket healing in the chitosan-γPGA hydrogel (C-PGA) group at various time points post-extraction. A. After 1 wk of healing following tooth extraction, no radiographically detectable calcification was present in the extraction sockets. B.Histologically, the C-PGA graft filled most of the socket. Inflammatory cell infiltration was observed around the C-PGA graft, and some woven bone formation extended from the socket-wall surface into the provisional collagen fibril matrix. C.After 2 wk, some radiographically detectable calcification was present in the extraction socket. D. Most regions of the extraction socket were filled with trabecular bones, which formed between the peripheral and central regions of the socket. The C-PGA was almost degraded. E. After 4 wk, socket healing continued to be characterized by the marked formation of new bone, radiographically. F. No residual material was identified, and prominent calcification filled the extraction sockets. Trabecular bone formation was identified in the peripheral and central portions of the extraction socket. G. After 6 wk, calcified tissue was clearly visible throughout the extraction socket, and trabecular bone filled almost the entire socket. Arrows indicate the length of the extraction socket (scale bar in B, D, F, and H is 1.0 mm).

The radiographic and histopathological findings regarding socket healing in the C-PGA group are shown in Fig. 6. At 1 wk post-extraction, most of the extraction sockets treated with C-PGA remained filled with C-PGA. Inflammatory cell infiltration was observed around the graft, and new bone formation between the peripheral and central regions of the socket was clearly visible. At 2 wk following extraction, most of the graft was degraded. Granulocyte infiltration was visible at the periphery of the graft. In approximately 50% of the sockets, trabecular bones formed between the peripheral and central regions of the sockets. Thus, the extraction sockets treated with C-PGA sustained more extensive new bone formation than that of the other study groups. At 6 wk post-extraction, the sockets were almost entirely filled with trabecular bone and the degree of calcification was more extensive than that observed at previous time points.

### Radiographic assessment of socket healing

**Figure 7:**
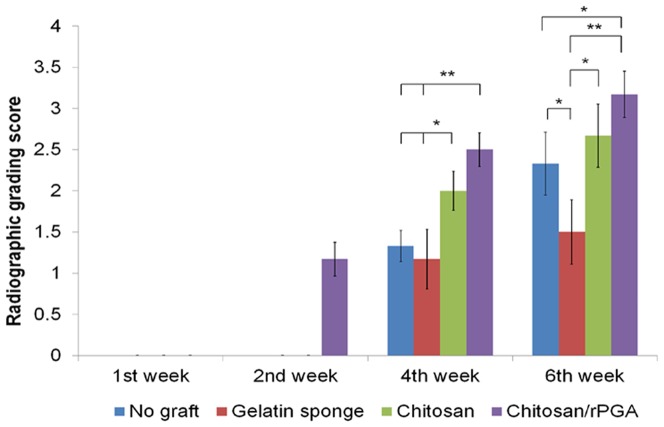
Radiographic assessment of socket healing. At 1 wk post-extraction, all of the sections exhibited radiolucency in the extraction sockets, indicating that no obvious calcification occurred during 1 wk. After 2 wk of healing, sockets treated with C-PGA demonstrated a small degree of radiopacity, whereas the other groups did not. At 4 and 6 wk post-extraction, all of the groups exhibited various degrees of calcification, and the highest degree of radiopacity was observed in the sockets treated with C-PGA, which were followed by those treated with neat chitosan, no treatment, and the gelatin sponge, in the order of decreasing degrees of radiopacity (* and ** indicate significant differences (P < 0.05) between the groups labeled, with each).

Radiography was used to demonstrate socket calcification following tooth extraction. In general, the degree of calcification was enhanced as the radiopacity increased in the sockets of the rats used in this study (Fig. 7). After 1 wk of healing, all of the sections exhibited radiolucency in the extraction sockets, indicating that no obvious calcification occurred. After 2 wk of healing, the extraction sockets treated with C-PGA exhibited a small degree of radiopacity, whereas other groups did not. At 4 and 6 wk post-extraction, all of the groups exhibited various degrees of calcification and the highest degree of radiopacity was observed in the sockets treated with C-PGA, followed by those treated with chitosan, the control extraction sockets, and those treated with the gelatin sponge, in the order of decreasing degrees of radiopacity.

### Histopathological assessment of socket healing

**Figure 8:**
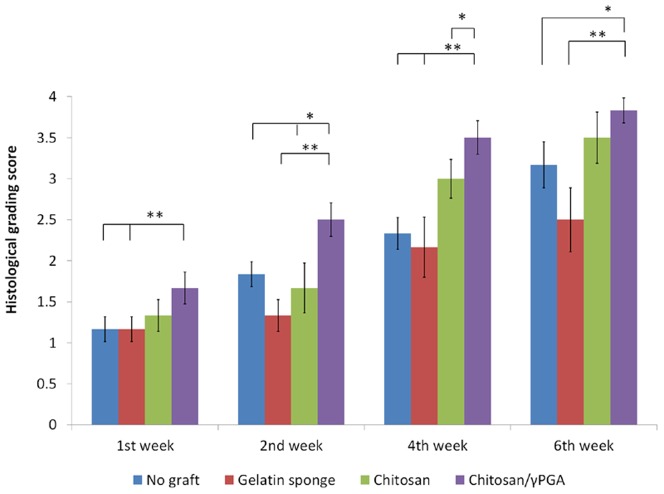
Histopathological assessment of various grafts used in socket healing. At both 1 and 2 wk post-extraction, the sections exhibited significantly greater amounts of new bone formation in the sockets of the C-PGA group than those in the chitosan, gelatin sponge, and control groups. After 4 wk of healing, at least 2 or more sites of new bone or ossicle formation were visible in the chitosan and C-PGA groups, whereas significantly less bone formation was observed in the gelatin sponge and control groups. After 1, 2, 4, and 6 wk, the sockets treated with C-PGA received the highest qualitative scores for bone formation (*P* < 0.05), followed by those treated with the neat chitosan, the control extraction sockets, and those treated with the gelatin sponge, in the order of decreasing scores. (* and ** indicate significant differences (*P* < 0.05 and *P* < 0.01, respectively) between the groups labeled, with each).

The results of the histopathological assessment are shown in Fig. 8. At both 1 and 2 wk post-extraction, the sections exhibited significantly more new bone formation in the sockets treated with C-PGA than in the other study groups. After 4 wk of healing, at least 2 or more sites of new bone or ossicle formation were visible in the sockets treated with neat chitosan or C-PGA, whereas significantly less bone formation was observed in the sockets treated with the gelatin sponge and in the control sockets. At 1, 2, 4, and 6 wk post-extraction, the sockets treated with C-PGA received the highest qualitative scores for bone formation (P < 0.05), followed by those treated with the neat chitosan, the control extraction sockets, and the gelatin sponge, in the order of decreasing scores.

### Morphometric analysis of the ground sections

**Figure 9:**
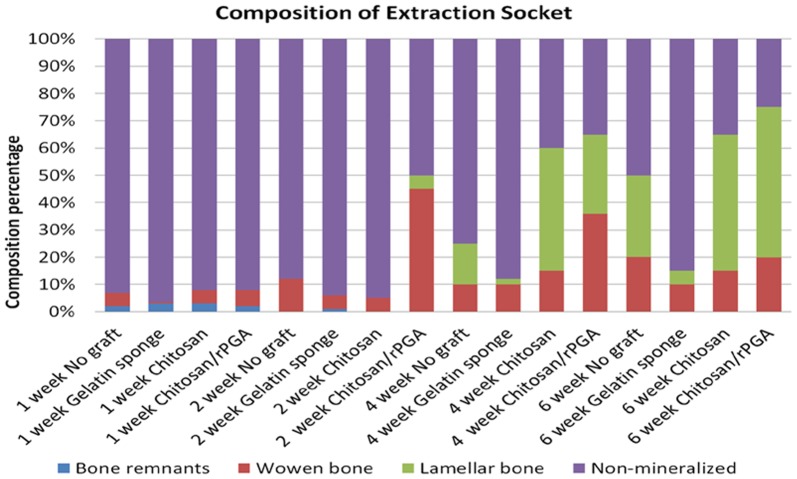
Histogram of the results derived from the morphometric analysis of the ground sections. Assessments were performed at 1, 2, 4, and 6 wk post-extraction. Proportions of woven bone, lamellar bone, non-mineralized tissue, and bone remnants in the extraction socket are represented. At 1 wk post-extraction, all of the study groups exhibited similar proportions of new bone formation, except for the gelatin sponge group, which exhibited the lowest proportion of woven bone formation. At 2 wk post-extraction, the sockets treated with C-PGA demonstrated a dramatic increase in the proportion of new bone formation. Woven bone formation increased up to 44.6% and the evidence of lamellar bone formation was clearly visible, whereas all of the other study groups displayed similar proportions of new bone formation. At 4 wk post-extraction, although the patterns of new bone formation were dissimilar between the 2 groups, the sockets treated with C-PGA and those treated with neat chitosan exhibited higher proportions of new bone formation than those of the other study groups. At 6 wk, the sockets of all of the study groups exhibited over 50% new bone formation, except for those treated with the gelatin sponge, which exhibited the least woven and lamellar bone formation. The sockets treated with C-PGA exhibited the highest proportion of new bone formation at 6 wk post-extraction, demonstrating 55.1% and 19.9% increases in lamellar bone and woven bone, respectively, during the same period.

The morphometric analysis of the proportions of woven bone, lamellar bone, non-mineralized tissue, and bone remnants in the extraction sockets are shown in Fig. 9. At 1 wk post-extraction, all of the study groups exhibited similar proportions of new bone formation, except for the gelatin sponge group. At 2 wk post-extraction, the sockets treated with C-PGA demonstrated a dramatic increase in the proportion of new bone formation. Woven bone formation increased up to 44.6% and the evidence of lamellar bone formation was clearly visible, whereas all of the other study groups exhibited similar proportions of new bone formation.

At 4 wk post-extraction, the sockets treated with C-PGA or neat chitosan exhibited higher proportions of new bone formation than that of the other study groups. At 4 wk post-extraction, the sockets treated with gelatin sponge and the control sockets exhibited similar proportions of woven bone formation. However, the sockets treated with the gelatin sponge exhibited significantly less lamellar bone formation than that of the control sockets.

After 6 wk of healing, the sockets of all of the study groups exhibited over 50% new bone formation, except for those that were treated with the gelatin sponge. The sockets treated with C-PGA exhibited the highest proportion of new bone formation, demonstrating 55.1% and 19.9% increases in lamellar bone and woven bone formation, respectively. However, the formation of woven bone decreased during the same period.

### Morphometric analysis of the decalcified sections

**Figure 10:**
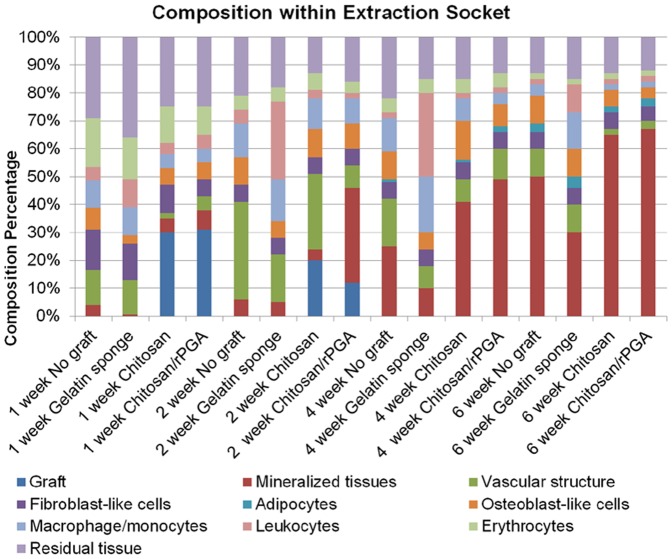
Histogram of the results derived from the morphometric analysis of the decalcified sections (1000× magnification) comprising the extraction sockets. After 1 wk of healing, similar mineralization processes were exhibited in the tissues of all of the study groups, except in those treated with the gelatin sponge, which exhibited significantly less mineralized tissue. At 2 wk post-extraction, the C-PGA group demonstrated a dramatic increase of up to 34% in the proportion of mineralized tissue, whereas the other study groups maintained similar proportions of mineralized tissue. At 4 and 6 wk post-extraction, the sockets treated with C-PGA and those treated with chitosan exhibited a significantly higher proportion of mineralized tissues than the other study groups. The gelatin sponge group displayed the least amount of mineralized socket tissues. After 1 wk of healing, a higher proportion of vascular structure was observed in the sockets of the control group and those of the gelatin sponge group. At 2 wk post-extraction, the C-PGA group exhibited the lowest proportion of vascular structure, compared with the vascular structure observed in the other groups. At 4 and 6 wk post-extraction, the sockets treated with C-PGA and those treated with neat chitosan exhibited a lower proportion of vascular structures than that of the other study groups. The sockets treated with gelatin sponge exhibited the highest proportion of vascular tissues. Although the proportion of leukocytes present in the sockets was similar among all of the study groups after 1 wk of healing, the gelatin sponge group exhibited a significantly higher proportion of leukocytes than that of the other groups.

The morphometric analysis of the decalcified sections is shown in Fig. 10. In the chitosan and C-PGA groups, a higher proportion of the grafts remained at 1 wk post-extraction than at the other time points. However, the grafts began to degrade at 2 wk following extraction. No graft remained at 4 and 6 wk post-extraction. The gelatin sponge group exhibited significantly less mineralized tissue than that of the other groups at 1 wk. After 2 wk of healing, the sockets treated with C-PGA demonstrated a dramatic increase of up to 34% in the proportion of mineralized tissue. At 4 and 6 wk post-extraction, the C-PGA and chitosan groups exhibited a significantly higher proportion of mineralized tissues than that of the other study groups.

After 1 wk of healing, a higher proportion of vascular structure was observed in the control and gelatin sponge-treated sockets than that observed in the other groups. At 2 wk post-extraction, the C-PGA group exhibited the lowest proportion of vascular structure. At 4 and 6 wk post-extraction, the C-PGA and chitosan groups exhibited a lower proportion of vascular structures than that of the other study groups.

The proportion of adipocytes present in the alveolar sockets of all of the study groups was not obvious at 1 and 2 wk post-extraction. At Week 4, the chitosan, C-PGA, and control groups demonstrated increased proportions of adipocytes, which continued to increase after 6 wk of healing, and a measurable increase in the proportion of adipocytes in the sockets treated with the gelatin sponge was also observed at 6 wk post-extraction.

The presence of osteoblast-like cells was observed in all of the study groups at 1 wk post-extraction. At 2 wk post-extraction, the highest proportion of osteoblast-like cells was observed in the chitosan and C-PGA groups, indicating that more rapid new bone formation occurred in these groups than in the other groups. After 4 and 6 wk of healing, the proportion of osteoblast-like cells decreased in the sockets treated with chitosan or C-PGA, whereas the proportion of osteoblast-like cells increased during this period in the sockets treated with the gelatin sponge and remained essentially unchanged in the control sockets.

Macrophages and monocytes were observed in all of the sockets at 1 wk post-extraction. At 2 and 4 wk post-extraction, the proportion of macrophages and monocytes decreased in the chitosan and C-PGA-treated groups. At 4 and 6 wk post-extraction, all of the study groups demonstrated significant decreases in the proportion of macrophages and monocytes, except for the group treated with the gelatin sponge.

The proportion of fibroblast-like cells and leukocytes after 1 wk of healing was significantly higher in the control and gelatin sponge groups than that in other groups.

## Discussion

We previously described the properties of chitosan and γ-PGA in the introduction. The positive charge of chitosan is soluble in a low pH environment and possesses antibacterial abilities. γ-PGA is also water-soluble and its anonic nature allows it to form PEC hydrogel with chitosan in a biologically suitable pH range. The antibacterial activity of γ-PGA against both E. Coli and S. aureus has been reported in previous studie [11].

Our previous study demonstrated that chitosan-γ-PGA PECs adhere more smoothly to the wound surface than neat chitosan does [12]. These PECs can simultaneously absorb exudates and allow the wound to remain sufficiently moist. Chitosan-γ-PGA PECs exhibit strong hydrophilicity and excellent tissue affinity with γ-PGA, and consequently produce an advantageous environment for wound healing. In this study, we used the same logic to prove that chitosan-γ-PGA PEC hydrogel is capable of accelerating new bone formation.

We proposed a rat model combined with histological and radiographic evaluations to demonstrate the superior new bone growth that occurred in extraction sockets treated with C-PGA, compared with the new bone growth that occurred in sockets that received other treatments. One advantage of the animal model was that the section plane was easily determined and greatly facilitated the comparison of the results regarding the contralateral extraction socket. Although a canine model has been used to evaluate socket healing following extraction [20]–[23], the proposed rat model may allow for more sensitive comparisons between the control and treatment groups in studies on extraction socket healing.

We used radiographic and histomorphological analyses to evaluate simultaneously the healing efficacy of the various treatments used. In most of the groups, the early events of new bone formation were detected using the histological analysis as early as 1 wk post-extraction. However, only the sockets treated with C-PGA demonstrated radiographic changes after 4 wk of healing, indicating that administering treatment with C-PGA facilitated the early detection of calcification. The ground and decalcified sections were used for the histomorphological analysis of the extraction sockets in this study. As described by Abrahamsson et al. [20], the analysis of the ground sections provided an overview of the various phases of tissue formation, whereas the analysis of the decalcified sections enabled a more detailed study of the events involved in bone tissue modeling and remodeling. The initially empty socket was filled with a coagulum and granulation tissue that was replaced by a provisional matrix. The process of bone formation started at 1 wk post-extraction in the sockets treated with C-PGA. The newly formed bone that was present on the lateral wall of the socket and the sporadic newly formed woven bone were visible in the area adjacent to the graft. In addition, lamellar bone and bone marrow replaced this primary bone after 2 wk of healing. In the C-PGA group, lamellar bone formation was observed earlier and more initial woven bone was detected than those that occurred in the other groups. Establishing and maintaining socket healing is a dynamic process. Although we observed similar resorption and appositional events among the sockets of the other groups, the rate and degree of bone formation was significantly greater in the sockets treated with C-PGA.

Because chitosan contains antiseptic properties and is biocompatible and biodegradable, it has been described as a potential scaffold material for bone grafting [24]. Bumgardner et al. also demonstrated that chitosan exhibits osteoconductive and enhanced wound healing properties, which suggests that chitosan may be useful as a bioactive coating to improve the ossification of orthopedic and craniofacial implants. Therefore, chitosan-derived substances may be useful in dentistry and periodontology. [25]

The chemical structure of chitosan is similar to that of hyaluronic acid, which has also been reported to exhibit wound-healing properties. Muzzarelli et al. [26]–[29] described the main characteristics of chitin-derived wound healing materials as biodegradability, biocompatibility, and the capacity to promote hyaluronan synthesis. The biological activity of hyaluronan may involve the formation of chito-oligomers that stimulate various cells from which monomers are phosphorylated and incorporated into hyaluronan, keratan sulphate, chondroitin sulphate, or components of the extracellular matrix and connective tissue.

The healing processes favored by chitosan-based materials that were previously examined were macrophage activation, cytokine production using macrophages and fibroblasts, anti-inflammatory action, angiogenesis stimulation, granulation, and scar formation. Based on in vitro data, Klokkevold et al. suggested that chitosan potentiates the differentiation of osteoprogenitor cells that facilitate bone formation [2]. Therefore, chitosan-based materials may be valuable for biomedical procedures, such as the treatment of leg ulcers, the application of skin substitutes, and the regeneration of bone, nerve, and meniscus tissues.

The prominent new bone growth in the sockets treated with C-PGA was significantly more extensive at 2 wk post-extraction than that observed in the other groups. Therefore, the combination of chitosan and γPGA may enhance bone healing by accelerating the healing process during early healing events. In the demineralized sections of the sockets treated with C-PGA, the relative amount of mineralized tissue increased up to 34%. In the ground section analysis, sockets treated with C-PGA also exhibited some lamellar bone formation (6.5%) as early as 2 wk post-extraction. At 6 wk post-extraction, the sockets treated with C-PGA contained a much higher proportion of lamellar bone than that of the other study groups. By using the proposed rat model, we demonstrated that C-PGA is a possible bone scaffolding material for healing extraction sockets.

In addition to increasing the quantity of new bone growth, administering treatment with C-PGA also improved the quality of the newly formed bone, as evidenced by the extensive lamellar bone formation that occurred. Although the scanning electron microscopy results revealed similar architecture and topography, such as pore size and arrangement, the differences in the chemical composition of the chitosan and C-PGA grafts may be responsible for the differences in osteoinduction observed for the 2 graft materials. Previous studies have reported on the superiority of C-PGA in other applications, such as dermatological grafting [12], [30] and mucosa repair [31]. Hsieh et al. proposed that the γ-PGA-chitosan composite matrices are extremely promising biomaterials for tissue engineering applications, such as environments for cell attachment and proliferation, because they exhibit greater hydrophilicity, more favorable cytocompatibility, and a more extensive mechanical structure, compared with that of chitosan alone [32]. Hsieh et al. also fabricated a novel, porous composite scaffold by blending chitosan and γPGA, and demonstrated that the composite scaffold was effective for sustaining and controlling the release of rhBMP-2 which may induce bone and cartilage regeneration. Therefore, C-PGA-based composite materials may be helpful in a broad range of applications used in bone-regenerative therapies [33].

## Conclusion

We proposed an animal model to evaluate the efficacy of C-PGA as a scaffolding material for facilitating new bone growth in alveolar sockets following tooth extraction. The results indicated that extraction sockets treated with C-PGA exhibited significantly earlier as well as greater amounts of new bone formation than treatment with a gelatin sponge or chitosan alone did, suggesting that C-PGA may be a useful scaffold for facilitating new bone growth in the extraction sockets.
